# Approach-Avoidance Behavior in the Empathy for Pain Model as Measured by Posturography

**DOI:** 10.3390/brainsci11111426

**Published:** 2021-10-28

**Authors:** Harold Mouras, Thierry Lelard

**Affiliations:** 1 UR-UPJV 4559, Laboratoire de Neurosciences Fonctionnelles et Pathologies, UFR de Médecine, Université de Picardie Jules Verne, 80000 Amiens, France; 2 UR-UPJV 3300, Adaptations Physiologiques à l’Exercice et Réadaptation à l’Effort (EA 3300), UFR des Sciences du Sport, Université de Picardie Jules Verne, 80000 Amiens, France; thierry.lelard@u-picardie.fr

**Keywords:** empathy, pain, posturography, social neuroscience, affective neuroscience, mental simulation

## Abstract

The interrelation between motor and emotional processes has been a recurrent question since several decades in the scientific literature. An interesting experimental technique to explore this question is posturography which assess the modulation of human postural control. In an emerging scientific field, this technique has been used to explore the reaction of the body in different emotional conditions. However, among available studies, some inconsistencies appear. In this brief report, we want to show how a widely used experimental model, i.e., empathy for pain, allowed in several study to provide comprehensive understanding elements on the postural correlates of socioemotional information processing. In particular, the role of mental simulation is discussed.

## 1. Introduction

The investigation of the link between emotional and motor processes fits into a long scientific tradition already theorized by Darwin [1]. Among different theories, the link between motor and emotional responses involves a degree of automaticity with emotional stimuli triggering automatic responses for survival, for example. Several studies [2,3,4,5,6] have demonstrated that emotional processing influences behavior in all its components, such as central, cognitive and motor. A well-known working model to explore the link between emotion and motricity is a biphasic model arguing that behavioral responses are led by motivational circuits with pleasant appetitive stimuli inducing approach-type responses and unpleasant defensive stimuli inducing withdrawal-type responses [7,8,9,10,11].

The interrelation between motor and emotional processes can be explored through a simple motor response, i.e., posture. Postural control can act as a framework for perception and action with respect to the external world by allowing one to interact with the environment. Postural control can be measured through instruments such as posturography which quantifies the body sway, notably by the displacement of the center of pressure (COP) along the anteroposterior dimension (meaning the direction between the subject and any visual/emotional/social target). The COP represents the point of application of the ground reaction force. Consequently, its position can be modified by distributing plantar pressures [12], where a positive value shows an approach toward a visual target, whereas a negative value shows a withdrawal. As compared to other techniques such as EEG and EMG that explore other sides of the motor correlates of socioemotional information processing, posturography is distinguished by two original aspects: (i) the very peripheral nature of the measurement collected, which certainly leads to a certain latency in its implementation, but which makes it possible to measure the motor output stage, testifying to the effective modulation of posture (both unconscious and conscious); (ii) the fact that the participant is not asked to perform any task, thus making it possible to record the processes at work in a more ecological environment than that which would be constrained by an experimental task.

Until now, several studies have used posturography to explore the posturographic correlates of emotional processing, indexing the interrelations between motor and emotional processes [13]. Across these studies, the postural correlates of aversive stimuli processing appear complex, with studies reporting withdrawal behavior in response to unpleasant visual stimuli [14] and others reporting freezing responses [15,16,17]. Some studies reported the same freezing strategy in response to positive stimuli [18], leading some authors to say that postural responses seem to depend more on arousal than on valence [9].

A well-recognized experimental model to study this link is empathy for pain which uses classical visual stimuli depicting painful or non-painful scenes (pictures depicting painful or non-painful situations involving the hands or feet and selected from a more extensive database, previously validated; [19]) and compares responses induced in participants by these conditions on different levels. In this perspective paper, we want to shed light on our main results obtained in studies that used pain simulation and empathy for pain to broadly explore the postural modulation induced by emotional processing. To simplify our discussion, we will mainly focus on the anteroposterior position of the COP as an index of postural modulation.

## 2. Empathy for Pain to Explore Approach-Avoidance through Posturography in Socioaffective Neuroscience

Almost one decade ago, we conducted a preliminary study which was the first to implement posturography within the experimental model of empathy for pain [20]. Participants were asked to imagine themselves in the painful or non-painful scenes presented for empathy for pain tasks. Basically, and in all the other studies mentioned here, posturography was mainly used to focus on the modulation of the anteroposterior position of the body in response to stimuli pertaining to different experimental conditions. An anterior modulation of the posture was interpreted as a reduction of the distance between the subject and the target (conscious or unconscious; i.e., an approach-type behavior) whereas a posterior modulation was interpreted as an increase of the distance between the subject and the target (conscious or unconscious; i.e., an avoidance-type behavior). This study allowed us to demonstrate a differential modulation of postural control by the valence of the presented stimuli as indexed by another postural index, the anteroposterior path, which was significantly shorter (*p* < 0.05) for painful situations (M = 148.0 ± 33.4 mm) as compared to non-painful ones (158.2 ± 38.7 mm). This tendency did not confirm our primary hypothesis of a withdrawal-type behavior in response to painful (i.e., negatively valenced) stimuli.

**Figure 1:**
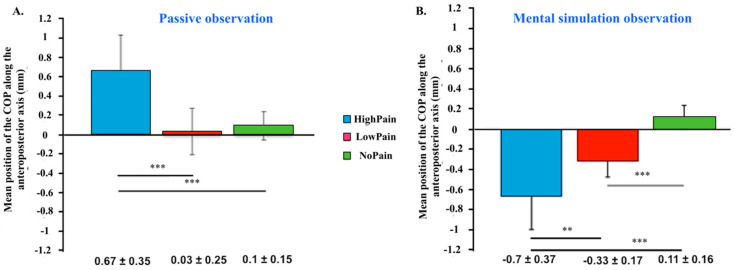
Mean position (±SEM) of the center of pressure (COP) along the anteroposterior axis of visual pain stimuli presentation in passive condition (first run, **A**) and mental simulation condition (second run, **B**). Significance codes: 0 ‘***’ 0.001 ‘**’ 0.01. First published in Beaumont A, Granon S, Godefroy O, Lelard T, Mouras H. Impact of pain intensity on postural response to visual exposure to painful stimuli: modulation by embodiment processes. *Experimental Brain Research* 2021; doi:10.1007/s00221-021-06102-y.

This study was interesting because we first shed light on motor control and pain simulation through spontaneous movement expressed by automatic postural responses. However, the results of this first study were modulated by critical methodological limitations. In particular, we were unable to distinguish the respective effect on postural control of mental simulation and the level of pain represented because we did not include a “passive vision” condition in our experimental conditions during which no self-projection instructions were given to the participants. To better understand the effect of mental simulation of painful situations, two other studies were conducted. In both studies, we incorporated both “passive vision” and “mental simulation” conditions that allowed us to disentangle the mental simulation and pain effects on postural control [20,21]. In the most recent study [22], we included more participants (39) than in previous studies [20,21] (31 participants). Importantly, this study was the first to demonstrate significant mean differences in COP_AP_ position (Figure 1). First, when comparing the postural responses recorded in response to painful rather than non-painful visual stimuli, the mean position of the COP along the anteroposterior axis was significantly different both in “passive observation” and “mental simulation” conditions. Secondly, the results were fascinating when comparing the “passive observation” and “mental simulation observation” conditions, with an approach-type behavior becoming a withdrawal-type behavior towards painful stimuli. This effect was reported either for low or high-painful stimuli. Third, this study was also the first to report modulation of this effect by the level of depicted pain.

## 3. The Question of Time to Apprehend the Complexity of the Responses

**Figure 2:**
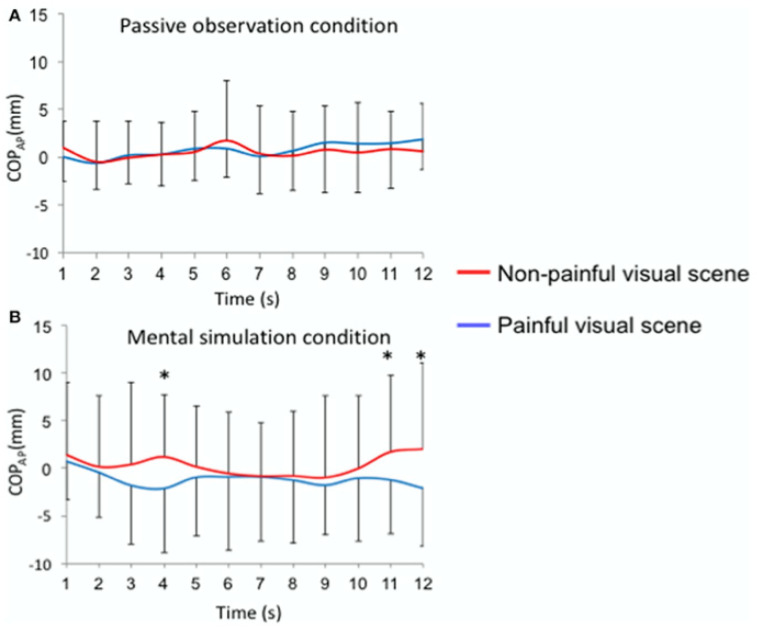
Time course of the anteroposterior position of the COPAP for each visual stimulus (painful vs. non painful visual scenes). Mean (±SEM) sliding window (1 s): (**A**) passive observation condition; (**B**) mental simulation condition, * *p* < 0.05. First published in Lelard T, Godefroy O, Ahmaidi S, Krystkowiak P, Mouras H. Mental simulation of painful situations impacts posture and psychophysiological parameters. *Frontiers in Psychology* 2017; 8: 2012.

As mentioned above, a study [21] was dedicated to alleviating the limitations of a possible confound between a valence and a simulation modulatory effect on the postural control exerted during socioemotional processing. Although analyses did not allow us to report mean differences across conditions, the exploration of the temporal course of the response made visible complex differences that were notably understandable by assessing only the average level of response over the 12 s period of visual stimuli presentation (Figure 2).

As depicted in Figure 2A, the extraction of the temporal course of the postural indexes is interesting to put in relation with the results obtained in [6] on the mean values of the same indexes. This importance of time has been shown in posturographic studies and other aspects of socioemotional neuroscience [23]. Taking into account the temporal dynamic of the responses, it was possible to report a significant posterior displacement of the COP in the anteroposterior direction (indexing a withdrawal) in response to pain as compared to the non-painful visual scenes at the 4th, 11th and 12th seconds of the stimulus presentation. These differences were demonstrated in the mental simulation condition but not in the passive observation condition.

## 4. How Coherent Are Subjective and Objective Measures of Approach-Avoidance?

Substantial progress in [22] was made in collecting subjective ratings regarding approach and avoidance tendency with the different experimental conditions and the recording of postural responses. Whereas an “instruction” effect was found for the posturographic correlates of painful visual stimuli processing with an approach-type behavior within the “passive observation” condition becoming an avoidance-type behavior (in accordance with our primary hypothesis) when considering the “mental simulation observation” condition. Interestingly, this modulatory effect is not reported for the subjective rating of “evoked avoidance”, with a high level of evoked avoidance for both “passive observation” and “mental simulation observation” conditions.

## 5. Discussion

The use of posturography to investigate the peripheral correlates of socioemotional information processing remains recent. Although a widely used functional context in socioaffective neuroscience, empathy for pain has only been used in the emerging field of the exploration of the posturographic correlates of socioaffective processes. However, the preliminary results shed light on essential discussion angles that have broader implications for socioaffective neuroscience. Here, our purpose is not to give exhaustive reminders of the discussion arguments developed in previous publications [20,21,22], but to point out two specific aspects of our results that: (i) raise broader questions for socioaffective neuroscience; and (ii) will be at the foreground of the future needed studies focusing on the posturographic correlates of empathy for pain and socioemotional processes.

### 5.1. Dichotomy between Subjective and Objective Measures of Approach-Avoidance

We reported in our results for the same dimension, i.e., approach-avoidance, sometimes opposite patterns when comparing objective and subjective responses. For example, in response to passively viewing painful stimuli compared to neutral ones [22], participants reported a high-avoidance subjective feeling, whereas posturography assessed an approach tendency towards the painful stimuli. This raises the link between objective behavioral responses (such as posturography) and subjective responses (recorded from participants through Likert-type scales and predefined subjective scales). As we are reminded in [22], this difference between subjective and objective measures has been observed across different situations within socioaffective contexts: (i) sexual motivation where one can observe a difference between a non-perception of genitals whereas an objective response can be measured [24]; (ii) parenting behavior (for example, in response to emotional vocalization of the baby) in which motor behavior is not regulated by the pleasantness (regulating the subjective tendency to approach) of the “stimulus” but by the urgency of the stimulus [25]; (iii) pollution perception [26] posturographic correlates in which we were able to record primary approach toward polluted scenes judged as unpleasant.

When looking at this interesting dichotomy, what is the primary interpretation? To us, this raises the question of the level of consciousness of the postural correlates: this seems to be the first approach towards painful stimuli, which could be the key to building a good perception of the situation to develop a conscious and coherent behavioral response, later becoming a withdrawal after integration of all the relevant information.

### 5.2. The Effect of Mental Simulation

When looking at the modulatory effect of mental simulation on the postural correlates of painful stimuli viewing, our results demonstrate that when passively viewing painful stimuli, there is an approach towards painful stimuli. When manipulating mental simulation (i.e., when giving the participants the instruction to imagine themselves in the depicted scenes), we reported an approach-type behavior towards painful stimuli becoming inverted in an avoidance-type behavior under mental simulation. Through the lens of embodied cognition and motor theory of empathy, it is tempting to interpret this modulatory effect of mental simulation as an index of an increased embodiment of the situation. However, in the absence of an accurate, objective measure of embodiment and under the perspectives of the multiple psychological and neural processes involved in embodiment, we would say that mental simulation could be a key lever of embodiment but also that numerous other components must occur to provide an exemplary embodiment of the situation.

When looking at our data, we want to shed light on two main points:

- Figure 1 demonstrated an approach-type behavior towards painful stimuli within the passive observation condition as compared to an avoidance-type behavior towards these stimuli within the mental simulation condition;
- Figure 2 showed that to take into consideration the effect of time allowed us to demonstrate for the COP-AP position significant differences when comparing painful visuals and non-painful ones:
  ∘ only in the mental simulation condition as compared to the passive observation condition;
  ∘ which were demonstrated at two distinct stages of the temporal course: a relatively “early” one (4 s after simulation) and a later one (11 and 12 s after stimulation).

Taken together, it seemed reasonable to us to propose the hypothesis of a temporal modulation of the anteroposterior position of the COP in emotional/motivational conditions mediated by mental simulation; that could be the support for the difference between an “early instinctive” postural response and a “later reasoned” one.

One of its primary interests is its feasibility in providing peripheral correlates of approach-avoidance-type behavior when considering posturography. However, the recorded signal has relatively poor explanation power and does not infer the cognitive process linked to these motor correlates. Recent studies have provided insights on the central correlates of approach-avoidance-type behavior [27]: (i) these central correlates have been explored through electroencephalography (EEG) with relatively good temporal resolution; and (ii) in our studies, we underlined the importance of studying the temporal dynamic of the posturographic response [23]; the simultaneous recording of the postural correlates and the neural ones by EEG will be one of the major perspectives of the field.

Critical perspectives also appear when considering the importance of mental simulation. As explained above, mental simulation has been demonstrated to exert a modulatory effect on the posturographic correlates of empathy for pain. Recently, within a more “societal” framework, for the postural correlates of pollution perception (unpublished data), we could not reproduce the modulatory effect of mental simulation. Whereas we observed a significant modulatory effect of stimuli valence (polluted vs. clean) on the anteroposterior position of the COP, this effect was not modulated by mental simulation instructions given to the participants as we observed in the framework of empathy for pain. Henceforth, we can evoke several perspectives regarding the mental simulation effect on posturographic correlates. To simultaneously measure postural and brain responses within a theoretical framework such as empathy for pain would allow one to explore the modulatory effect of mental simulation on the posturographic and neural correlates of the same emotional processing. This question is of significant importance as we noted a different effect of mental simulation on subjective ratings and posturographic correlates, bringing up questions on the nature of the cognitive mechanism on which mental simulation could act. Moreover, fine-grained manipulations of the mental simulation process (in terms of intensity, means of its creations, etc.…) would allow the exploration of the respective parametric modulations of postural, neural and subjective responses by mental simulation.

We should also pay particular attention to the high variability of posturographic responses to the simulation of a painful situation. Interindividual variability might be linked to individual pain-related fear status [28]. This perspective is essential when considering our recent results showing the importance of pain intensity perception on posturographic correlations [22]. This can also be considered through the lens of clinical practice with multiple pathological conditions influencing pain perception.

Another perspective is the use of posturography beyond the framework of empathy for pain. As mentioned, we recently used posturography to explore the interaction between motor and emotional processes during pollution perception (unpublished data). Moreover, recently we used posturography in the field of alcohol-addictive behavior [29], demonstrating a distinct pattern of spontaneous movements that differentiate “abstainers” and “relapsers”.

## Data Availability

Details about data availability are mentionned in each original article.
